# One‐level mini‐open pedicle subtraction osteotomy for treating spinal kyphosis in patients with ankylosing spondylitis

**DOI:** 10.1186/s12891-021-03974-7

**Published:** 2021-01-22

**Authors:** Yu Wang, Chunde Li, Long Liu, Longtao Qi

**Affiliations:** Department of Orthopaedics, Peking University First Hospital, 100034 Beijing, China

**Keywords:** Ankylosing spondylitis, Kyphosis, Pedicle subtraction osteotomy, Vertebral decancellation, Mini‐open, Minimally invasive

## Abstract

**Background:**

To report a mini-open pedicle subtraction osteotomy (PSO) technique, to summarize the 2-year follow-up results of 25 patients, and to describe a modified operating table which allows the osteotomy to be closed in a more controllable manner.

**Methods:**

We retrospectively reviewed the records of patients with AS who received one-level mini-open PSO between July 2015 and January 2018. The 25 patients with complete medical records and 2-year radiographic follow-up were included in the analysis. Estimated blood loss, operation time, incision length, complications, bed rest period, and length of hospitalization were extracted from the medical records and recorded.

**Results:**

The mean age of the 25 patients (22 males and 3 females) was 39.5 years. The average global kyphosis(GK) decreased from 70.2° before surgery to 22.3° after surgery; the average C7- sagittal vertical axis (C7-SVA) decreased from 15.5 cm before surgery to 5.1 cm after surgery; the average pelvic incidence(PT) decreased from 37.8° before surgery to 22.5° after surgery. The average length of the incision was 10.2 cm. The average surgical time was 263.0 min, the average estimated blood loss was 840.0 ml, and the average time to mobilization was 4.1 days.

**Conclusions:**

The current report shows that one-level PSO can be performed through an incision of about 10 cm. The one-level mini-open PSO could be superior to traditional PSO surgery with respect to cosmetic outcomes. Further comparative studies are necessary to evaluate the current and conventional techniques.

## Background

Ankylosing spondylitis (AS) is a chronic inflammatory disease that can lead to ossification of the joints and ligaments of the spine. AS-related thoracolumbar kyphotic deformity is a disabling condition that affects more than 30 % of patients with AS. Osteotomy is extensively used to correct AS-related kyphosis, and 2 main types of osteotomy are used: Smith-Petersen osteotomy (SPO) and pedicle subtraction osteotomy (PSO) [1–7].

Osteotomy surgeries for correcting AS-related kyphosis are usually associated with long incisions, extensive soft tissue destruction, long operative time, and significant blood loss [8, 9]. Major complications have been reported in up to one-third of patients with AS-related kyphosis who receive an osteotomy procedure [9–14]. As such, developing less invasive methods of osteotomy is a topic of the current research. One such method is that described by Wang and Chou: a mini-open approach for PSO which combines percutaneous screw placement and a 3-column osteotomy [15–17]. In our practice, we also developed a method that is performed in a mini-open fashion. By improving operating procedure and using a modified operating table, we have found that it is possible to perform a PSO via a 10-cm incision.

Since 2015, we have performed a one-level mini-open PSO for select patients with AS. The purpose of this report is to describe the mini-open PSO technique, and to summarize the 2-year follow-up results of 25 patients.

## Methods

### Patient selection

We retrospectively reviewed the records of patients with AS who received one-level mini-open PSO between July 2015 and January 2018. The 25 patients with complete medical records and 2-year radiographic follow-up were included in the analysis. Estimated blood loss, operation time, incision length, complications, bed rest period, and length of hospitalization were extracted from the medical records and recorded.

### Radiographic follow‐up

**Fig. 1:**
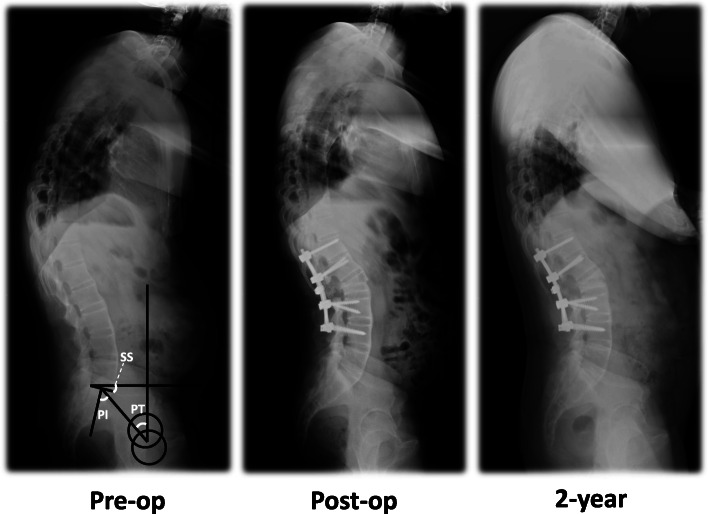
Preoperative, immediate postoperative and 2-year postoperative standing anteroposterior and lateral digital radiographs were reviewed

Radiographic measurements were performed using the radiology department picture archiving and communication system. Preoperative, immediate postoperative and 2-year postoperative standing anteroposterior and lateral digital radiographs were reviewed (Fig. 1).

### Surgical technique

**Fig. 2:**
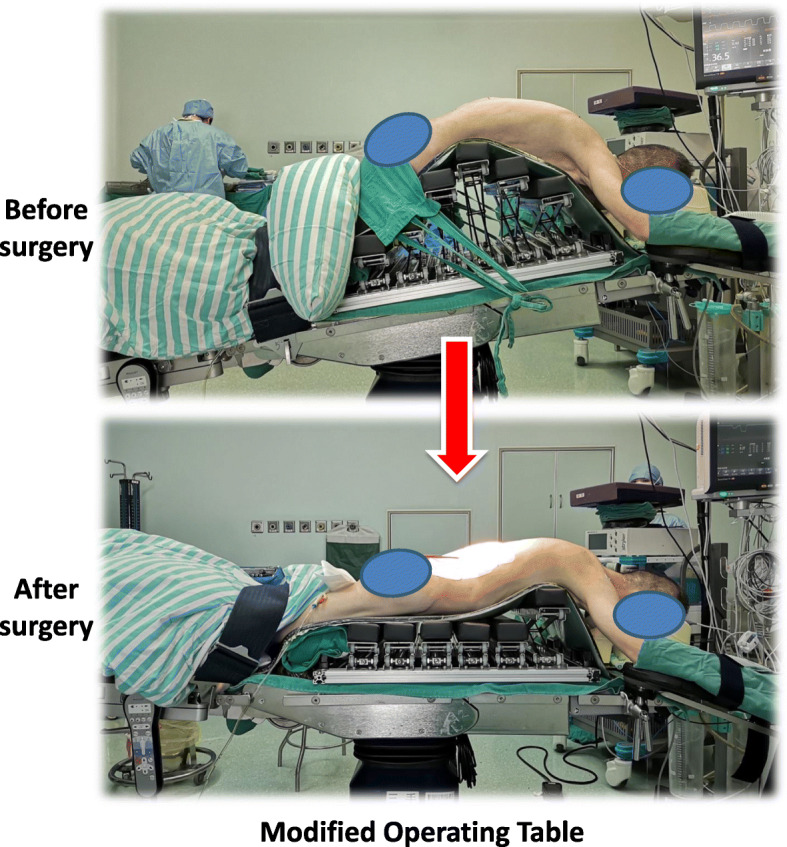
The modified operating table consists of 6 supports, and the height of each support can be adjusted separately. This allows the shape of the operating table to be adjusted to fit that of the patient. In addition, adjusting the supports allows the osteotomy to be closed in a more controllable manner so that vertebral subluxation can be prevented

A modified operating table that allows adjustment of the spinal alignment of AS patients during surgery is used for one-level mini-open PSO. After the PSO has been completed, the kyphotic deformity of an AS patient can be gradually corrected by manipulating the operating table (Fig. 2). This method increases control of the correction, and decreases the operative risks.

Typically, a one-level mini-open PSO begins with placement of screws at L1 and L3, followed by a PSO at L2, and then finally placement of screws at T12 and L4. All of the procedures are performed through one incision.

**Fig. 3:**
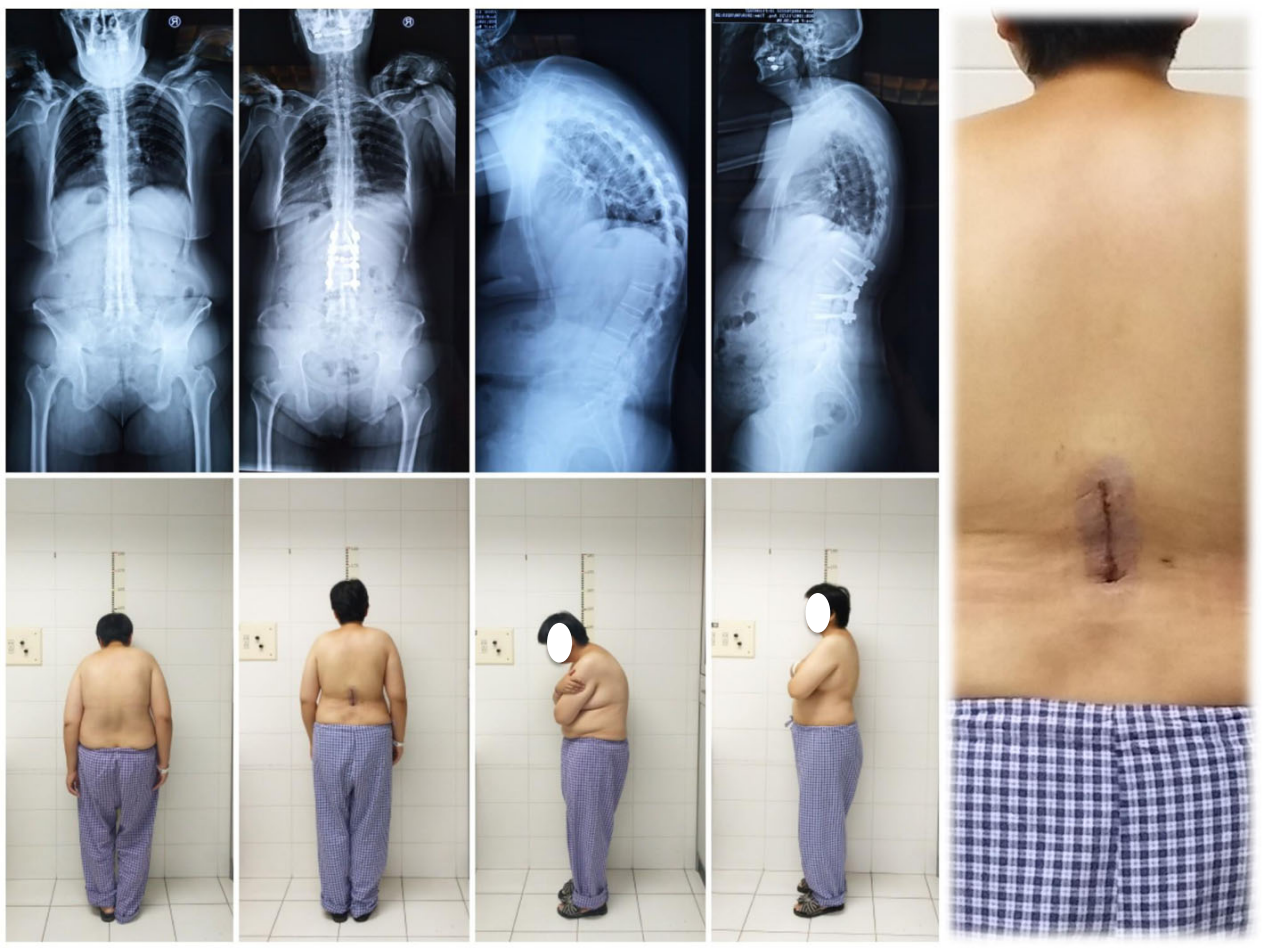
A 37-year-old female with AS and thoracolumbar kyphosis. The global kyphosis angle decreased from 72.3 preoperatively to 24.7r after surgery. The length of the incision was 9.8 cm

Specifically, a midline skin incision of about 10 cm beginning at L1 and ending at L3 is made. The paravertebral muscles are detached and the laminae are exposed bilaterally, as in traditional open surgery. After exposure, 4 pedicle screws (multi-axial, long-arm) are inserted at the level of L1 and L3, and screw position is checked by fluoroscopy. If all of the screws are deemed to be well-placed, an L2 PSO is performed. Immediately prior to completion of the PSO, 2 short rods are inserted and locked to the screws to prevent vertebral subluxation. Once the PSO is finished, the modified operating table is manipulated and the nuts on the L1 level screws are loosened. With gradually manipulation of the operating table the kyphotic deformity is corrected accordingly. When the V-shaped osteotomy is closed completely, the nuts are locked again. As a result, the posterior portion of the spine is shortened around 5 cm, and T12 and L4 move closer towards L2. Thus, the facet joints of T12 and L4 can be reached through the existing incision (Fig. 3).

**Fig. 4:**
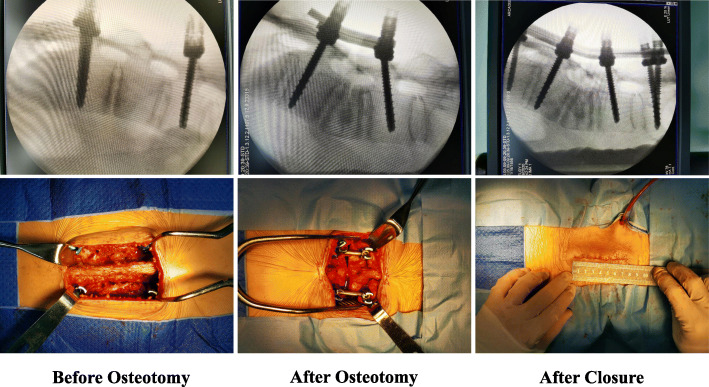
A midline skin incision of about 8 cm was made. After exposure, 4 pedicle screws (multi-axial, long-arm) were inserted at the level of L1 and L3, and screw position was checked by fluoroscopy. When the V-shaped osteotomy was closed completely, 4 screws were inserted at the level of T12 and L4, and then 2 long rods were instrumented

The facet joints are then exposed, and 4 screws are inserted at the level of T12 and L4 and fluoroscopy is performed to check their positions. If all the screws appear to be well-placed, the 2 short rods are replaced by 2 long rods. Somatosensory evoked potentials are monitored during the whole procedure. A drainage tube is inserted, and the incision is closed in layers. The drainage tube is removed when the daily drainage amount is < 100 ml. Typically, the patient is mobilized 3–4 days after surgery, and discharged 7–8 days after surgery (Fig. 4).

## Results

The mean age of the 25 patients (22 males and 3 females) who underwent one-level mini-open PSO was 39.5 years(range: 27–56 years).

### Radiographic follow‐up

The average global kyphosis(GK) decreased from 70.2° before surgery to 22.3° after surgery; the average C7- sagittal vertical axis (C7-SVA) decreased from 15.5 cm before surgery to 5.1 cm after surgery; the average pelvic incidence(PT) decreased from 37.8° before surgery to 22.5° after surgery (Table 1).

**Table 1:** Radiographic follow-up

Radiographic parameters	Preoperative	Postoperative	2-year follow-up	Final correction
TK (°)	41.8±14.8	43.3±13.5	42.1±12.8	0.3±9.2
LL(°)	-0.9±18.4	-40.4±16.3	-39.4±15.9	38.5±13.6
GK(°)	70.2±20.1	22.3±18.2	24.1±17.6	46.1±11.5
C7-SVA (cm)	15.5±5.8	5.1±7.5	6.3±6.8	10.6±5.6
PT(°)	37.8±10.7	22.5±7.5	23.1±6.9	14.7±7.8
PI (°)	41.7±9.8	42.9±9.9	42.7±9.7	1±5.8
SS(°)	4.2±10.3	20.4±9.8	21.6±8.9	17.4±8.9

*TK* Thoracic kyphosis, *LL* Lumbar lordosis, *GK* Global kyphosis, *SVA* Sagittal vertical axis, *PT* Pelvic tilt, *PI* Pelvic incidence, *SS* Sacral slope

### Intraoperative and postoperative results

The average length of the incision was 10.2 ± 1.2 cm (Table 2). The average surgical time was 263.0 ± 50.4 min, the estimated blood loss was 840.0 ± 417.1 ml, and the average time to mobilization was 4.1 ± 1.3 days.

**Table 2:** Intraoperative and Postoperative Results

	One-level PSO (n=25)
Length of incision(cm)	10.2±1.2
Number of fusion levels	4
Estimated blood loss(ml)	840.0±417.1
Operative time(min)	263.0±50.4
Time to mobilization(days)	4.1±1.3

*PSO* Pedicle subtraction osteotomy

### Complications

No deaths, complete paralysis, or vascular complications occurred, and no patient required a revision surgery. No implant-related complication was found. 3 patients experienced cerebrospinal fluid leak due to a dural tear.

## Discussion

PSO is widely used for treating AS-related kyphosis. The procedure was first described by Thomasen in 1985. PSO involves removal of posterior spinal elements, and a transpedicular wedge osteotomy of the vertebral body. When the wedge osteotomy is closed, the kyphotic deformity is corrected accordingly. As reported by many other authors, the mean correction achieved by one-level PSO ranges from 30° to 45° [2, 18–20].

### Can PSO be less invasive?

PSO is one of the most invasive spine surgeries, and as such a topic of current research is how to make the procedure less invasive. Wang and Madhavan described a technique for treating degenerative kyphoscoliosis using a mini-open PSO combined with percutaneous pedicle screws [15]. Chou reported 2 patients treated with the same technique [16]. The 2 patients, both with flat-back syndrome, underwent percutaneous fixation above and below the PSO, and the PSO was performed in a mini-open fashion. Charles [21] reported a 30-year-old female who had AS-related kyphosis who was treated with a combined open and percutaneous approach.

**Fig. 5:**
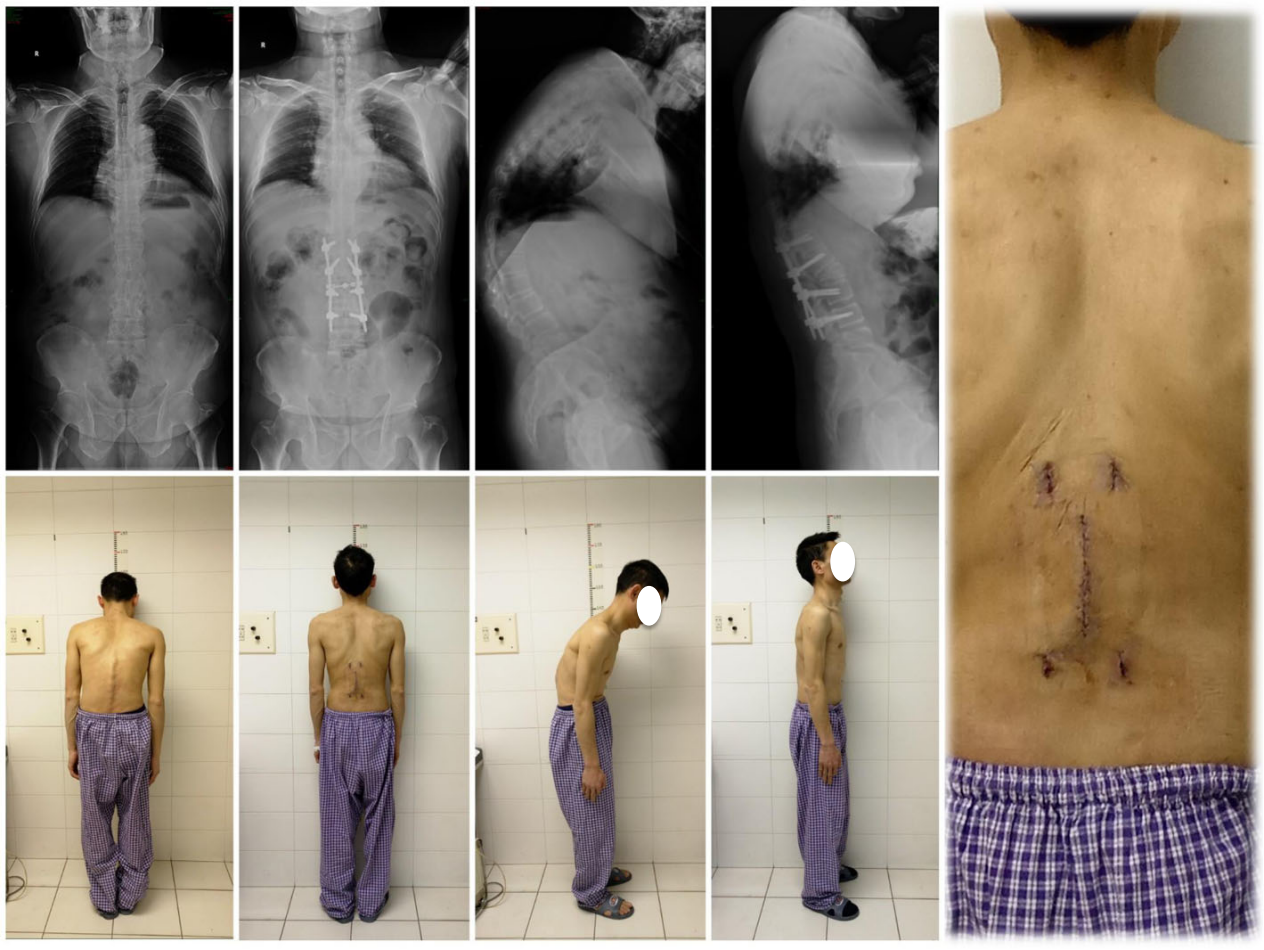
Prior to development of the current technique, we used a technique combining percutaneous screw placement and mini-open PSO, which required multiple incisions

Prior to the development of the technique described herein, we used a technique similar to that described above for treating AS-related kyphosis. The combination of percutaneous screw placement and mini-open PSO significantly decreased the length of the incision (Fig. 5).

As we advanced our technique, we realized that it was possible to alleviate the use of percutaneous screws. By improving the operating procedure, both screw placement and PSO can be done through one incision; this is because some screws can be inserted after PSO, rather than before PSO as in a traditional PSO surgery. When the posterior part of spine is shortened as the osteotomy is closed, more facet joints can be reached through the already existing incision. For example, a mini-open PSO begins with screw placement at L1 and L3, followed by a PSO at L2, and concludes with screw placement at T12 and L4. All the manipulations can be done through the same incision. In addition, the use of a modified operating table makes closing the osteotomy more controllable and less difficult, and thus makes the whole procedure more feasible. The average length of the incision in the 25 patients was 10.2 cm, which is significantly shorter than that in traditional PSO surgery. As such, we believe that mini-open PSO surgery is superior to PSO surgery in terms of cosmetics.

### How effective is one‐level mini‐open PSO?

The average final correction of the 25 patients was 46.1°, which is considered an excellent outcome as most studies report corrections of around 40° (Table 3).

**Table 3:** Comparisons to the literature

Author	No./age(yr)	Preop GK(°)	Final correction(°)	Final SVA (cm)	Final PT(°)	Operation time(min)	Blood loss (ml)
Zhu(2012) [27]	31/36	73.7	39.9	7.7	N/A	N/A	1740
Xu(2015) [28]	37/37	N/A	38.4	5.8	29.2	232	1240
Liu(2015) [20]	55/34	75.3	43.6	N/A	N/A	381	2729
Hua(2017) [23]	12/39.5	75.2	38.7	10.8	44.5	327	1525
Qiao(2018) [24]	17/35.3	67.56	39.4	6.4	N/A	N/A	N/A
Qiao(2018) [24]	47/34.2	74.28	44.4	5.2	N/A	N/A	N/A
Xin(2019) [6]	339/34.8	55.8	44.6	5.2	N/A	253	537
Huang(2019) [26]	100/34.7	71.6	47.6	4.5	23.3	332	1821
Wang(2019) [5]	27/40.3	50.6	28.9	8.7	N/A	301	1452
Wang(2019) [5]	30/37	49.3	31.7	5.2	N/A	279	1150
Current study	25/39.5	70.2	46.1	4.9	23.1	263	840

*GK* Global kyphosis, *SVA* Sagittal vertical axis, *PT* Pelvic tilt, *N/A* Not applicable

In recent years, more attention has been paid to sagittal spinal alignment during the correction of spinal deformities [22–24]. Schwab et al. proposed that SVA, PT, and PI-LL mismatch are the 3 most important spinopelvic parameters that should be carefully considered in the preoperative planning for treating adult spinal deformities. To achieve good clinical outcomes, the realignment objectives have been reported to be an SVA < 50 mm, PT < 25°, and PI-LL equal to ± 9° [25]. More recently, Huang et al. reported that PT was the major radiographic contributor to ODI score at the last follow-up in patients with ankylosing spondylitis [26]. The optimal sagittal alignment parameters at the 2-year follow-up of adult spinal deformity patients who underwent one-level PSO were a PT < 24°, spinosacral angle (SSA) > 108°, T1 pelvic angle (TPA) < 22°, and spinopelvic angle (SPA) > 152°. The average PT of the 25 patients in this study was 23.1°, which can be considered a satisfactory outcome according to the aforementioned criteria.

### How many levels should be instrumented?

Usually, 4–6 segments are instrumented in a PSO surgery [27, 28]. In other words, pedicle screws are inserted into at least 2 segments above and below the osteotomy level. In the study by Zhu et al.[27], an average of 5.1 segments was instrumented. Huang et al. compared long and short instrumentation in patients with AS-related kyphosis [26]. Sixty-four patients who underwent one-level PSO were divided into a short-segment group (17 patients, 4.5 segments instrumented) and a long-segment group (47 patients, 7.4 segments instrumented). The authors concluded that both long- and short-segment instrumentations can ensure maintenance of kyphosis correction, without obvious loss of correction. The extension of fused segments might not avoid instrumentation-related complications such as PJK or rod breakage in patients without fully ossified thoracolumbar structures. All 25 patients in this study received 4-segment instrumentation, and no implant-related complications occurred during the 2-year follow-up. We believe that instrumentation of 4 segments (2 segments above and below the osteotomy level) should be sufficient for a one-level PSO, because immediate 3-column stability of spine can be achieved when the osteotomy is completely closed, and hence there is no need for a longer instrumentation.

### The modified operating table

In traditional PSO surgery, patients are placed prone on a regular operating table, which is flexed in a reverse V-shape. Once the PSO is completed, the operating table is hyperextended to a V-shape, during which the osteotomy is gradually closed and the kyphotic spinal deformity is corrected. For our procedure, we use a modified operating table which consists of 6 independent supports. The height of each support can be adjusted separately so that the shape of the operating table can be adjusted to fit that of the patient. Adjusting the supports allows the osteotomy to be closed in a more controllable manner so that vertebral subluxation can be prevented.

## Conclusions

The current report shows that one-level PSO can be performed through an incision of about 10 cm. The one-level mini-open PSO could be superior to traditional PSO surgery with respect to cosmetic outcomes. Further comparative studies are necessary to evaluate the current and conventional techniques.

YW designed the concept of this study and drafted the manuscript. LL collected the clinical data. LTQ and CDL analyzed and interpreted the data. All authors have read and approved the final manuscript.

## Data Availability

All data generated or analysed during this study are included in this published article [and its supplementary information files].

## Abbreviations

AS: Ankylosing spondylitis
PSO: Pedicle subtraction osteotomy
SPO: Smith-Petersen osteotomy
TK: Thoracic kyphosis
LL: Lumbar lordosis
GK: Global kyphosis
SVA: Sagittal vertical axis
PT: Pelvic tilt
PI: Pelvic incidence
SS: Sacral slope
